# A novel signature to guide osteosarcoma prognosis and immune microenvironment: Cuproptosis-related lncRNA

**DOI:** 10.3389/fimmu.2022.919231

**Published:** 2022-07-29

**Authors:** Mingyi Yang, Haishi Zheng, Ke Xu, Qiling Yuan, Yirixaiti Aihaiti, Yongsong Cai, Peng Xu

**Affiliations:** Department of Joint Surgery, HongHui Hospital, Xi’an Jiaotong University, Xi’an, China

**Keywords:** osteosarcoma, LncRNA, immunity, prognosis, cuproptosis

## Abstract

**Objective:**

Osteosarcoma (OS) is a common bone malignancy with poor prognosis. We aimed to investigate the relationship between cuproptosis-related lncRNAs (CRLncs) and the survival outcomes of patients with OS.

**Methods:**

Transcriptome and clinical data of 86 patients with OS were downloaded from The Cancer Genome Atlas (TCGA). The GSE16088 dataset was downloaded from the Gene Expression Omnibus (GEO) database. The 10 cuproptosis-related genes (CRGs) were obtained from a recently published article on cuproptosis in *Science*. Combined analysis of OS transcriptome data and the GSE16088 dataset identified differentially expressed CRGs related to OS. Next, pathway enrichment analysis was performed. Co-expression analysis obtained CRLncs related to OS. Univariate COX regression analysis and least absolute shrinkage and selection operator (LASSO) regression analysis were used to construct the risk prognostic model of CRLncs. The samples were divided evenly into training and test groups to verify the accuracy of the model. Risk curve, survival, receiver operating characteristic (ROC) curve, and independent prognostic analyses were performed. Next, principal component analysis (PCA) and t-distributed stochastic neighbor embedding (t-SNE) analysis were performed. Single-sample gene set enrichment analysis (ssGSEA) was used to explore the correlation between the risk prognostic models and OS immune microenvironment. Drug sensitivity analysis identified drugs with potential efficacy in OS. Real-time quantitative PCR, Western blotting, and immunohistochemistry analyses verified the expression of CRGs in OS. Real-time quantitative PCR was used to verify the expression of CRLncs in OS.

**Results:**

Six CRLncs that can guide OS prognosis and immune microenvironment were obtained, including three high-risk CRLncs (AL645608.6, AL591767.1, and UNC5B-AS1) and three low-risk CRLncs (CARD8-AS1, AC098487.1, and AC005041.3). Immune cells such as B cells, macrophages, T-helper type 2 (Th2) cells, regulatory T cells (Treg), and immune functions such as APC co-inhibition, checkpoint, and T-cell co-inhibition were significantly downregulated in high-risk groups. In addition, we obtained four drugs with potential efficacy for OS: AUY922, bortezomib, lenalidomide, and Z.LLNle.CHO. The expression of LIPT1, DLAT, and FDX1 at both mRNA and protein levels was significantly elevated in OS cell lines compared with normal osteoblast hFOB1.19. The mRNA expression level of AL591767.1 was decreased in OS, and that of AL645608.6, CARD8-AS1, AC005041.3, AC098487.1, and UNC5B-AS1 was upregulated in OS.

**Conclusion:**

CRLncs that can guide OS prognosis and the immune microenvironment and drugs that may have a potential curative effect on OS obtained in this study provide a theoretical basis for OS survival research and clinical decision-making.

## Introduction

Osteosarcoma (OS) is the most common primary bone tumor originating from primitive mesenchymal cells (1). OS primarily affects long bones, with sarcoma cells forming immature bone or osteoid tissues (2). OS is the most common primary bone cancer in children and adolescents and the third most common bone cancer in adults, after chondrosarcoma and chordoma (2). OS mainly affects children and adolescents aged 10–30 years and has a bimodal age distribution, with the first peak at 15–19 years (8 cases/million/year) and the second at 75–79 years (6 cases/million/year) (3, 4). OS is highly malignant, with lesions that can spread throughout the body and metastasize to distant sites, most often to the lungs (5). Chemotherapy and surgical resection are the standard treatments for OS (6). Metastatic OS frequently recurs and has a poor prognosis, and combined chemotherapy treatment slightly improves OS compared with surgical resection alone (7). The 5-year survival rate for patients with localized OS is 80%, and the 5-year survival rate for patients with metastatic OS is 15%–30% (8). Over the past 20 years, despite many chemotherapy regimens, the OS rate has not improved significantly (9). No successful OS-targeted therapies have been developed (2). Therefore, exploring new targets and features to improve the clinical efficacy and survival of patients with OS is necessary.

The top international academic journal *Science* announced the existence of cuproptosis and the 10 discovered cuproptosis-related genes (CRGs): ferredoxin 1 (FDX1), lipolytransferase1 (LIPT1), lipoyl synthase (LIAS), dihydrolipoamide dehydrogenase (DLD), dihydrolipoamide S-acetyltransferase (DLAT), pyruvate dehydrogenase E1 subunit alpha 1 (PDHA1), pyruvate dehydrogenase E1 subunit beta (PDHB), metal regulatory transcription factor 1 (MTF1), glutaminase (GLS), and cyclin-dependent kinase inhibitor 2A (CDKN2A) (10). Cuproptosis is a new form of cell death that depends on copper ions and is regulated by cells (10). The mechanism by which cuproptosis causes cell death is distinct from all other known regulatory mechanisms of cell death, including apoptosis, ferroptosis, pyroptosis, and necrosis (10).

Copper-induced cell death is closely related to mitochondrial metabolism and the tricarboxylic acid (TCA) cycle (10). After interfering with mitochondrial function, the sensitivity of cells to copper ions was significantly altered. Copper-induced cell death requires mitochondrial respiration rather than ATP from glycolysis. Furthermore, copper ions are not directly involved in the electron transport chain but only play a role in the TCA cycle. TCA-cycle-related metabolites are significantly increased in copper-sensitive cells (10). Copper ions directly bind to fatty acylated components in the TCA cycle, resulting in the abnormal aggregation of fatty acylated proteins and loss of iron–sulfur cluster proteins, leading to protein toxic stress responses and ultimately cell death (10). Copper-induced fatty acylation and iron–sulfur cluster proteins in human cancer cells are conserved from bacterial to human evolution, suggesting that copper ionophores are naturally synthesized and display antibacterial activity and that microbes may contribute to cuproptosis (11).

FDX1 and proteoacetylation are key regulators of copper-ionophore-induced cell death (10). Elesclomol and diethyldithiocarbamate are structurally different copper ionophores. FDX1 not only reduces Cu^2+^ to the more toxic Cu^1+^ but is also a direct target of the copper ionophore elesclomol (12). Knockdown of seven CRGs (FDX1, LIPT1, LIAS, DLD, DLAT, PDHA1, and PDHB) rescued the cytotoxic effects of elesclomol and diethyldithiocarbamate (10). These seven CRGs are positively regulated during cuproptosis, and MTF1, GLS, and CDKN2A are negatively regulated during cuproptosis (10). FDX1 deletion confers resistance to various copper ionophores (disulfiram, NSC319726, thiram, 8-HQ, and Zn-pyrithione). Deletion of FDX1 and LIAS resulted in copper-induced cell death. Protein fatty acylation is a highly conserved post-translational lysine modification that occurs in only four enzymes: dihydrolipoamide branched chain transacylase E2 (DBT), glycine cleavage system protein H (GCSH), dihydrolipoamide S-succinyltransferase (DLST), and DLAT (13, 14). These enzymes are not only involved in regulating the metabolic complexes of carbon in the TCA cycle but also important components of the PDH complex (10). DLAT, PDHA1, and PDHB belong to the PDH complex, which is a protein target for fatty acylation. FDX1 is an upstream regulator of fatty acylation (10). FDX1 knockdown results in the accumulation of pyruvate and α-ketoglutarate and depletion of succinate, impairing protein fatty acylation by inhibiting the TCA cycle at PDH and α-ketoglutarate dehydrogenase (10). FDX1 knockout resulted not only in the loss of protein fatty acylation but also a marked decrease in cellular respiration at levels similar to those observed with LIAS deletion. DLAT and DLST can bind to copper ions, and when FDX1 deletion prevents protein fatty acylation, DLAT and DLST no longer bind to copper. Therefore, fatty acylation is necessary for copper ion binding (10). Copper ions directly bind to and induce oligomerization of fatty acylated DLAT, and the toxic production of fatty acylated proteins upon exposure to copper ionophores is partially mediated by abnormal oligomerization (10). Furthermore, depletion of the intracellular natural copper chaperone glutathione resulted in copper-dependent cell death, which was associated with decreased fatty acylation due to the attenuation of FDX1 and LIAS and increased DLAT oligomerization (10).

FDX1 and the abundance of fatty acylated proteins are highly correlated with various human tumors, and cell lines with high levels of fatty acylated proteins are sensitive to cuproptosis, suggesting that copper ionophore therapy could target tumors with this metabolic profile (10). A study found that OS was related to the TCA cycle (15). A meta-analysis study in an OS mouse model found that many key metabolites and most amino acids in glycolysis and TCA cycles were elevated during rapid tumor growth, possibly because of the high energy requirements and the conversion of anabolic processes during tumor proliferation (15). Serum metabolism studies in an OS mouse model of lung metastasis have shown reduced carbohydrate and amino acid metabolism but elevated lipid metabolism associated with tumor metastasis (15). Therefore, studying the correlation between OS and cuproptosis to explore the therapeutic targets of OS is necessary.

Studies have identified pyroptosis- and autophagy-related genes that can predict OS prognosis by using risk prognostic models (16, 17). In this study, a novel OS prognosis model was established to explore cuproptosis-related lncRNAs (CRLncs) that can guide OS prognosis and immune microenvironment. The model was effective in predicting the long-term prognosis of patients with OS.

## Materials and methods

### Data download and arrangement

The OS transcriptome data and clinical data of 86 cases were downloaded from The Cancer Genome Atlas (TCGA) database (https://portal.gdc.cancer.gov/) and included mRNAs and lncRNAs. The clinical data were sorted to include futime, fustat, sex, age at diagnosis in days, metastatic/non-metastatic, primary tumor site, and specific tumor site. The GSE16088 dataset, including 6 normal tissue samples and 14 OS tissue samples, was downloaded from the Gene Expression Omnibus (GEO) database (https://www.ncbi.nlm.nih.gov/geo/).

### OS-related differentially CRGs

The 10 CRGs were intersected with genes in the OS transcriptome data to obtain OS-related CRGs. The limma package of R performed differential analysis on the GSE16088 dataset to obtain differentially expressed genes (DEGs). The screening criteria were *p* < 0.05 and |logFC| > 0.65 (18). OS-related CRGs and DEGs were intersected to identify the differentially expressed OS-related CRGs.

### Enrichment analysis

The clusterProfiler package of R was used to conduct the Kyoto Encyclopedia of Genes and Genomes (KEGG) enrichment analysis for OS-related differentially expressed CRGs, and the screening criterion was *p* < 0.05.

### OS-related CRLncs

The limma package of R was used to perform co-expression analysis of OS-related differentially expressed CRGs and lncRNAs in OS transcriptome data to obtain OS-related CRLncs, and the screening criteria were |Pearson correlation coefficient| > 0.4 and *p* < 0.001 (19).

### Construction of risk prognostic model

The survival package in R was used to obtain statistically significant (*p* < 0.05) CRLncs associated with OS prognosis through univariate COX regression analysis and calculate the hazard ratio (HR) value. The glmnet package in R was used to perform LASSO regression analysis to narrow the risk of overfitting and determine the optimal number of CRLncs involved in model building. The samples were divided into training and test groups to verify the accuracy of the model. A risk prognosis model was built for the total sample, training, and test groups.


$$\text{Risk Score}=\sum_{i=1}^{n}\left(lncrnaexp_{i}\times coef_{i}\right)$$

where n represents the number of OS prognosis CRLncs, i is the ith CRLncs, coef is the regression coefficient, and the expression of OS prognosis CRLncs is multiplied by the corresponding regression coefficient and accumulated to obtain the sample risk score (16). The samples in the total sample, training, and test groups were divided into high- and low-risk groups according to the median of the risk score.

### Validation of risk prognostic model

Risk curve analysis, survival analysis, receiver operating characteristic (ROC) curve analysis, and independent prognosis analysis were performed on the risk prognostic models of the total sample, training, and test groups, respectively. R was used to draw the survival status map and risk heatmap of the risk prognosis model and observe the differences in patient survival time and OS prognosis CRLncs in high- and low-risk groups (17). The survival and survminer packages in R were used to construct survival curves, and the survival, survminer, and timeROC packages in R were used to plot ROC curves. The survival package in R was used to perform independent prognostic tests through univariate and multivariate COX regression analyses to test whether the risk score can be used as an independent prognostic factor (20).

### Principal component analysis and t-distributed stochastic neighbor embedding analysis

Principal component analysis (PCA) and t-distributed stochastic neighbor embedding (t-SNE) analysis were performed on the risk prognostic model of the total sample group to observe whether the expression of the OS prognostic CRLncs involved in the model construction could distinguish patients in the high- and low-risk groups to test the accuracy of the model.

### Tumor microenvironment analysis

The limma and estimate packages of R were used to perform tumor microenvironment analysis on OS transcriptome data to obtain immune scores, stromal scores, and total scores for each OS patient (21). The limma and ggpubr packages of R were used to analyze whether immune, stromal, and total scores differed in the risk prognostic model of the total sample group (16).

### Single-sample gene set enrichment analysis

The GSVA, limma, and GSEABase packages in R were used to obtain enrichment scores for immune cells and immune function for OS transcriptome data. The limma, reshape2, and ggpubr packages in R were used to analyze the differences in immune cells and immune function in the risk prognosis model of the total sample group (16).

### Drug sensitivity analysis

The limma, ggpubr, and pRRophetic packages of R were used to perform drug sensitivity analysis to determine which drugs had different sensitivities in the risk prognostic model of the total sample group and to screen potential therapeutic drugs for OS, with *p* < 0.001 as the screening criterion.

### Cell culture

Human OS cell lines (HOS, 143B, and U2OS) and human normal osteoblast cell (hFOB1.19) were purchased from Wuhan Procell Life Science and Technology Co., Ltd. (Wuhan, China). Each cell line was cultured in its dedicated medium (Wuhan Procell Life Science and Technology Co. Ltd., Wuhan, China). Human OS cell lines were cultured at 37°C in an incubator with 5% CO_2_. The hFOB1.19 cells were cultured in a 34°C incubator with 5% CO_2_.

### Real-time quantitative PCR

Total RNA was extracted from OS cell lines and hFOB1.19 using TRIzol Reagent (Cat. No. P118-05, GenStar, Beijing, China) according to the manufacturer’s instructions. Total RNA was amplified by RT-PCR using SYBR Green Master Mix (Cat#: C0006, TOPSCIENCE, Shanghai, China) according to the manufacturer’s instructions, and the mRNA levels of CRGs and CRLncs were detected. The primer pairs were synthesized by Accurate Biology (Changsha, China), and the primer pairs are listed in **Table 1**. All samples were normalized to β-actin, and the 2^−ΔΔCt^ method was used to evaluate relative expression levels.

**Table 1:** Primer sequences for RT-qPCR.

Genes	Forward	Reverse
β-actin	TGGCACCCAGCACAATGAA	CTAAGTCATAGTCCGCCTAGAAGCA
LIPT1	GCTCTGAATGCTGTCCAACCC	GCAATGGTGATAGGCAGTAGTCC
DLD	GCCGACGACCCTTTACTAAGAAT	GGACCAGCAACTACATCACCAAT
PDHA1	CAGACCATCTCATCACAGCCTACC	CCTCCTTTCCCTTTAGCACAACCT
DLAT	GTTCCCATCGGAGCGATCAT	GCTGCTGAGGAATCCAGTGT
FDX1	CCTGGCTTGTTCAACCTGTCA	CCAACCGTGATCTGTCTGTTAGTC
CDKN2A	AGCACTCACGCCCTAAGC	TGACTCAAGAGAAGCCAGTAACC
AL645608.6	AGGTCCCACCATCCTCACAA	CGGACCCGAACTCTCAGATG
CARD8-AS1	CCTCAGCTGGAATGCCTTCAT	GGGTTACACACATTCTCGGC
AL591767.1	TGAGCTTAAACAAGCTTAGGAGTTA	CGCCCAGCTGGTTATTTTTGA
AC098487.1	CTGTAGAGAAGAGGAACCGTAGC	TGGTTGACCTAGAAATGGAAGGAA
UNC5B-AS1	GGGCCGGAGTTCCAATCAA	GCATTTCCCTGAGGCAGGAT
AC005041.3	TATCTTGCACCCACACACCC	TTATTGAGCAGGCCTCCGTG

### Western blotting

OS cells and hFOB1.19 were harvested with radioimmunoprecipitation assay (RIPA) buffer (Zhonghuihecai, Xi’an, China) and pelleted by centrifugation at 4°C for 15 min, and the supernatant was discarded. Next, 1/5 sodium dodecyl sulfate–polyacrylamide gel (SDS-PAGE) sample loading buffer, 5× (Beyotime, Shanghai, China) was added to the supernatant and heated in a 100°C metal bath for 10 min. The protein was separated on a 15% SDS-PAGE, transferred to a 0.22-μm polyvinylidene fluoride (PVDF) membrane (Millipore, USA), placed in 5% skimmed milk, blocked for approximately 2 h, and incubated with specific antibodies. Antibodies used were as follows: the DLAT (Cat. No. 13426-1-AP, 1:2,000), DLD (Cat. No. 16431-1-AP, 1:2,000), CDKN2A (Cat. No. 10883-1-AP, 1:1,000), and FDX1 (Cat. No. 12592-1-AP, 1:500) antibodies were purchased from Proteintech (Wuhan, China); PDHA1 (Cat. No. bs-4034R,1:500) and LIPT1 (Cat. No. bs-18298R,1:500) were purchased from Bioss (Beijing, China); and β-actin (Cat. No. AC026, 1:100,000, ABclonal). The protein bands were enhanced using a chemiluminescent kit (Vazyme, Nanjing, China).

### Immunohistochemistry and hematoxylin–eosin staining

CRGs were experimentally verified by immunohistochemistry (IHC) staining. Three OS tissue samples and one normal osteogenic tissue sample were collected from patients at Honghui Hospital, affiliated with Xi’an Jiaotong University. None of the patients received anti-cancer treatment before tissue sample collection. All patients signed an informed consent form, and the study was approved by the hospital ethics committee.

Tissues were fixed and paraffinized before immunohistochemistry (IHC) staining. Slides were cut to a width of 5 μm, dewaxed, and rehydrated. Endogenous peroxidase was inactivated by treatment with 3% H_2_O_2_ for 10 min, followed by incubation with a 5% bovine serum albumin (BSA) blocking solution for 1 h at room temperature. The cells were treated with hydrogen peroxide for 10 min to inactivate endogenous peroxidases. The sections were incubated with the corresponding protein antibodies overnight at 4°C. Antibodies used were as follows: the DLAT (Cat. No. 13426-1-AP, 1:500), DLD (Cat. No. 16431-1-AP, 1:500), and FDX1 (Cat. No. 12592-1-AP, 1:200) antibodies were purchased from Proteintech (Wuhan, China); PDHA1 (Cat. No. bs-4034R, 1:100) and LIPT1 (Cat No. bs-18298R, 1:100) were purchased from Bioss (Beijing, China); and CDKN2A (Cat. No. GB111143, 1:500, Servicebio). The sections were then incubated with biotinylated goat anti-rabbit secondary antibody for 30 min at 37°C. Color development was performed using freshly prepared 3,3′-diaminobenzidine (DAB) reagent (Boster, Wuhan, China). IHC staining of each tissue section was performed by two independent pathologists.

### Statistical analysis

Statistical analysis and visualization were performed using R.4.1.2, GraphPad Prism v8.2.1 and SPSS 22.0. Experimental results were expressed as mean ± SD (standard deviation), and statistical significance was determined by one-way ANOVA. *p* < 0.05 was considered statistically significant. Each experiment was performed at least three times independently.

## Results

### OS-related differentially CRGs and enrichment analysis

**Figure 1:**
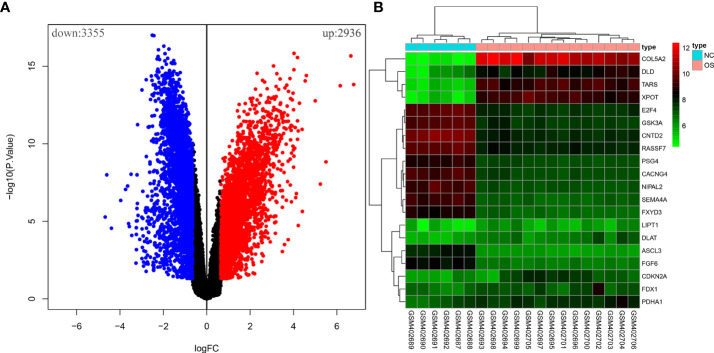
The differential analysis of the GSE16088 dataset. **(A)** Volcano plot of DEGs, red for high expression and blue for low expression. **(B)** Heatmap of DEGs, with high expression in red and low expression in green.

**Figure 2:**
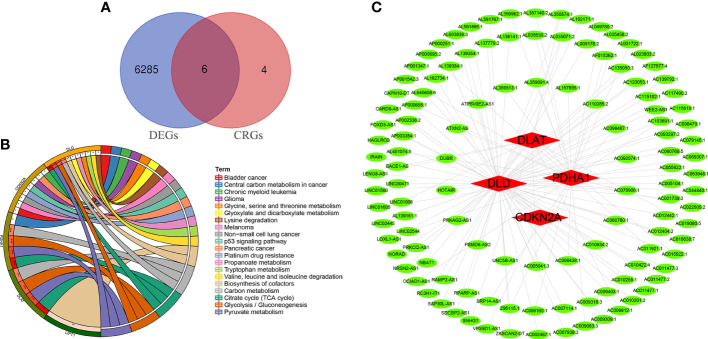
Differentially CRGs and CRLncs related to OS. **(A)** The intersection of DEGs and OS-related CRGs obtained OS-related differentially CRGs. **(B)** Pathway enrichment analysis of OS-related differentially CRGs. **(C)** By co-expression analysis, OS-related differentially CRGs yielded a total of 118 OS-related CRLncs.

Ten OS-related CRGs were identified. Difference analysis of the GSE16088 dataset obtained 6,291 DEGs: 2,936 upregulated DEGs and 3,355 downregulated DEGs. R was used to visualize volcano plots (**Figure 1A**) and heatmaps (**Figure 1B**). Finally, we identified six OS-related differentially expressed CRGs (**Figure 2A**). The six OS-related differentially expressed CRGs are highly expressed in OS. The six OS-related differentially CRGs were significantly enriched in the citrate cycle (TCA cycle); pyruvate metabolism; glycolysis/gluconeogenesis; carbon metabolism; biosynthesis of cofactors; glyoxylate and dicarboxylate metabolism; propanoate metabolism; glycine, serine, and threonine metabolism; bladder cancer; tryptophan metabolism; valine, leucine, and isoleucine degradation; lysine degradation; central carbon metabolism in cancer; melanoma; non-small cell lung cancer; platinum drug resistance; p53 signaling pathway; glioma; pancreatic cancer; and chronic myeloid leukemia (**Figure 2B**).

### Construction of risk prognostic model

**Figure 3:**
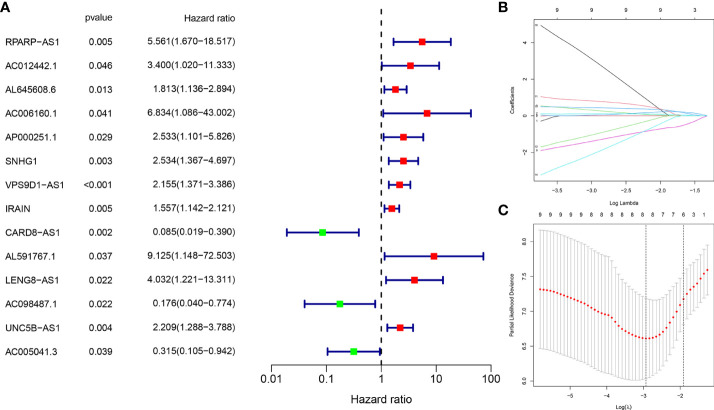
Construction of risk prognostic model. **(A)** Univariate Cox regression analysis obtained 14 candidate prognostic CRLncs for OS, including 11 high-risk CRLncs and three low-risk CRLncs. **(B)** LASSO regression analysis. **(C)** Selection of the optimal penalty parameter for LASSO regression.

A total of 118 OS-related CRLncs were identified by co-expression analysis (**Figure 2C**). Univariate COX regression analysis identified 14 CRLncs associated with OS prognosis, including 11 high-risk CRLncs (RPARP-AS1, AC012442.1, AL645608.6, AC006160.1, AP000251.1, SNHG1, VPS9D1-AS1, IRAIN, AL591767.1, LENG8-AS1, and UNC5B-AS1) and 3 low-risk CRLncs (CARD8-AS1, AC098487.1, and AC005041.3) (**Figure 3A**). According to the optimal penalty parameter (λ) value, the LASSO regression analysis determined that the optimal number of CRLncs participating in the model construction was 6 (**Figures 3B, C**). The risk score for each sample was obtained using the prognostic model formula. The total sample group was divided into a high-risk group (N = 47) and a low-risk group (N = 39). The training group was divided into high-risk (N = 21) and low-risk (N = 22) groups. The test group was divided into high-risk (N = 26) and low-risk (N = 17) groups.

### Risk prognosis models predict the prognosis of patients with OS

**Figure 4:**
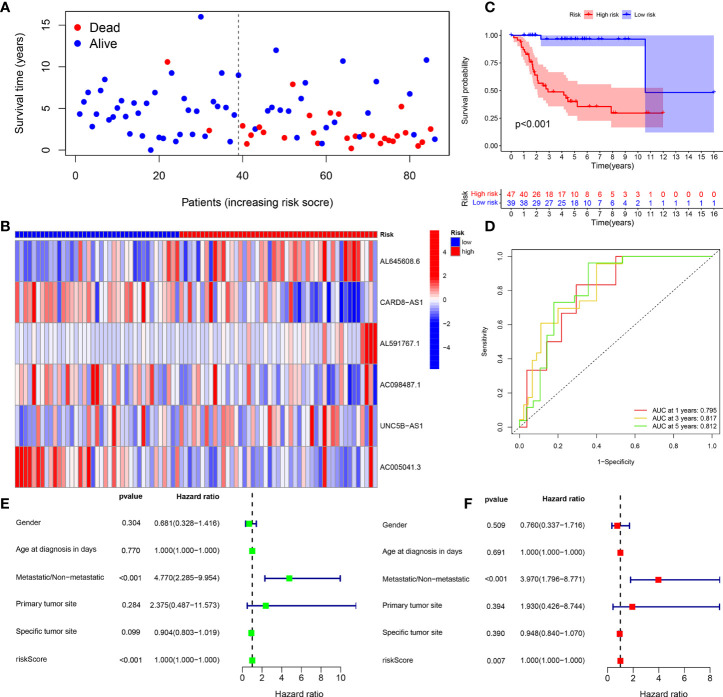
Total sample group. **(A)** Survival status map. **(B)** Risk heatmap. **(C)** Survival curve. **(D)** ROC curve. **(E)** Univariate COX regression analysis. **(F)** Multivariate COX regression analysis.

**Figure 5:**
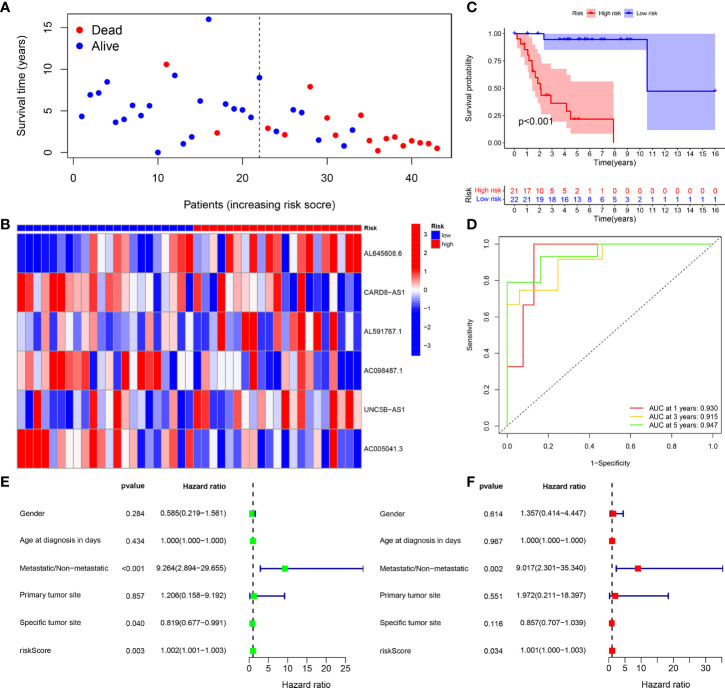
Training group. **(A)** Survival status map. **(B)** Risk heatmap. **(C)** Survival curve. **(D)** ROC curve. **(E)** Univariate COX regression analysis. **(F)** Multivariate COX regression analysis.

**Figure 6:**
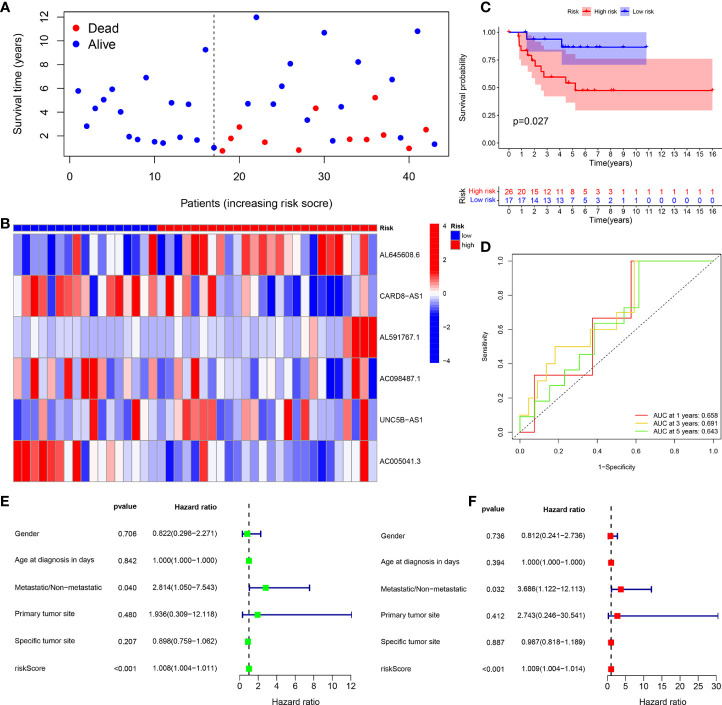
Test group. **(A)** Survival status map. **(B)** Risk heatmap. **(C)** Survival curve. **(D)** ROC curve. **(E)** Univariate COX regression analysis. **(F)** Multivariate COX regression analysis.

The survival status map of the total sample, train, and test groups showed that the mortality rate of patients from the low-risk group to the high-risk group gradually increased **(** **Figures 4A**, **5A**, **6A**). The risk heatmaps of the total sample group, training group, and test group showed that from the low- to the high-risk group, the expression levels of AL645608.6, AL591767.1, and UNC5B-AS1 gradually increased, which are high-risk CRLncs, and the expression levels of CARD8-AS1, AC098487.1, and AC005041.3 gradually decreased, which are low-risk CRLncs (**Figures 4B**, **5B**, **6B****)**.

The survival curves of the total sample group (*p* < 0.001), training group (*p* < 0.001), and test group (*p* = 0.027) showed that the survival of patients in the high- and low-risk groups was significantly different **(** **Figures 4C**, **5C**, **6C****)**. The ROC curve of the total sample group had a higher area under curve (AUC) at 1 year (AUC = 0.795), 3 years (AUC = 0.817), and 5 years (AUC = 0.812) (**Figure 4D**). The ROC curve of the training group had a higher AUC at 1 year (AUC = 0.930), 3 years (AUC = 0.915), and 5 years (AUC = 0.947) (**Figure 5D**). The ROC curve of the test group had a higher AUC at 1 year (AUC = 0.658), 3 years (AUC = 0.691), and 5 years (AUC = 0.643) (**Figure 6D**).

Univariate independent prognostic analysis of the total sample group showed that the risk score (*p* < 0.001, HR = 1.000) and tumor metastasis (*p* < 0.001, HR = 4.770) can be used as independent prognostic factors, which are high-risk factors (**Figure 4E**). Multivariate independent prognostic analysis of the total sample group showed that the risk score (*p* = 0.007, HR = 1.000) and tumor metastasis (*p* < 0.001, HR = 3.970) can be used as independent prognostic factors, which are high-risk factors (**Figure 4F**). Univariate independent prognostic analysis of the training group showed that both risk score (*p* = 0.003, HR = 1.002), tumor metastasis (*p* < 0.001, HR = 9.264), and specific tumor site (*p* = 0.040, HR = 0.819) can be used as independent prognostic factors, among which risk score and tumor metastasis were high-risk factors and specific tumor sites were low-risk factors (**Figure 5E**). Multivariate independent prognostic analysis of the training group showed that the risk score (*p* = 0.034, HR = 1.001) and tumor metastasis (*p* = 0.002, HR = 9.017) can be used as independent prognostic factors, which are high-risk factors (**Figure 5F**). Univariate independent prognostic analysis of the test group showed that the risk score (*p* < 0.001, HR = 1.008) and tumor metastasis (*p* = 0.040, HR = 2.814) can be used as independent prognostic factors, which are high-risk factors (**Figure 6E**). Multivariate independent prognostic analysis of the test group showed that the risk score (*p* < 0.001, HR = 1.009) and tumor metastasis (*p* = 0.032, HR = 3.686) can be used as independent prognostic factors, which are high-risk factors (**Figure 6F**). Our risk prognostic model indicated that the risk score and tumor metastasis could be independent prognostic factors for patients with OS, and both were high-risk factors.

**Figure 7:**
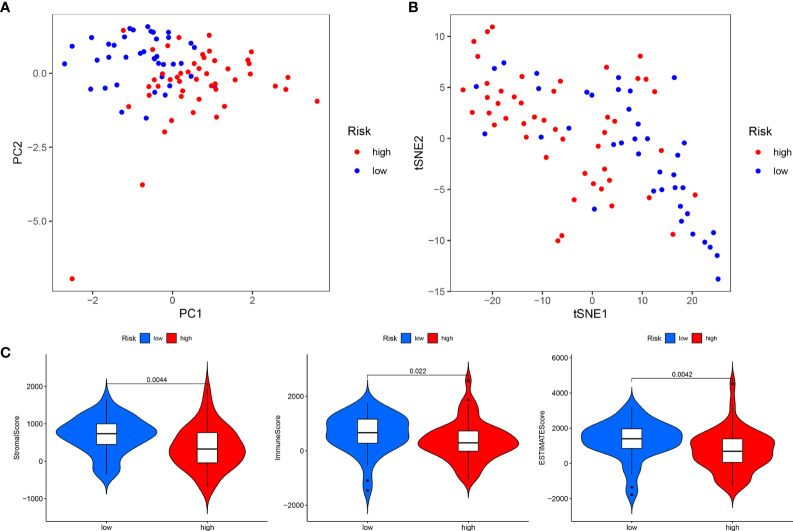
Total sample group. **(A)** Principal component analysis. **(B)** t-Distributed stochastic neighbor embedding analysis. **(C)** Differential analysis of tumor microenvironment.

In addition, PCA and t-SNE analyses revealed that the expression levels of OS prognostic CRLncs involved in model construction could significantly distinguish patients in the high- and low-risk groups, illustrating the accuracy of the model (**Figures 7A, B**). Thus, our risk prognostic model can well predict the survival of patients with OS.

### Risk prognostic models guide immune microenvironment of patients with OS

**Figure 8:**
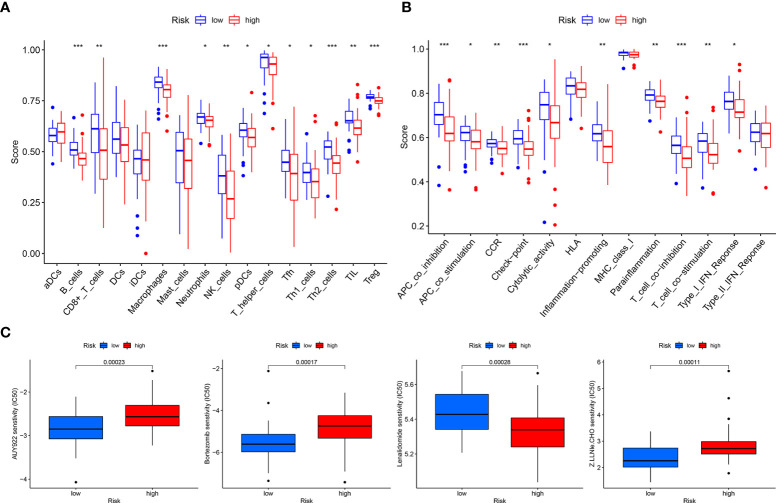
Total sample group. **(A)** Immune cell differential analysis for single sample gene set enrichment analysis. **(B)** Immune function differential analysis for single sample gene set enrichment analysis. **(C)** Drug sensitivity analysis.

The tumor microenvironment difference analysis showed differences in the stromal cell scores (*p* = 0.0044), immune cell scores (*p* = 0.022), and total scores (*p* = 0.0042) in the high- and low-risk groups, and the scores in the low-risk group were higher than those in the high-risk group (**Figure 7C**). Differential analysis of immune cells showed that B cells, macrophages, T-helper type 2 (Th2) cells, and regulatory T cells (Tregs) were significantly downregulated in the high-risk group (*p* < 0.001) (**Figure 8A**). Differential analysis of immune function showed that APC co-inhibition, checkpoint, and T-cell co-inhibition were significantly downregulated in the high-risk group (*p* < 0.001) (**Figure 8B**).

### Drugs with potential efficacy in OS

Drug sensitivity analysis revealed that AUY922 (*p* = 0.00023), bortezomib (*p* = 0.00017), lenalidomide (*p* = 0.00028), and Z.LLNle.CHO (*p* = 0.00011) showed significant sensitivity in the high- and low-risk groups. Patients in the low-risk group were more sensitive to AUY922, bortezomib, and Z.LLNle.CHO than patients in the high-risk group, and patients in the high-risk group were more sensitive to lenalidomide than patients in the low-risk group (**Figure 8C**).

### Validation of the expression of CRGs and CRLncs in OS

**Figure 9:**
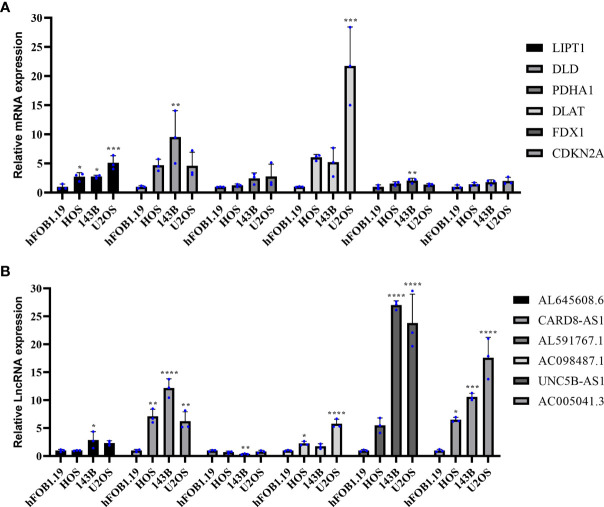
Validation of the mRNA expression level of CRGs and CRLncs in OS cell lines. **(A)** The mRNA expression level of CRGs. **(B)** The mRNA expression level of CRLncs. *p < 0.05, **p < 0.01, ***p < 0.001, ****p < 0.0001 each experiment was repeated three times.

To further assess the expression of CRGs and CRLncs, we selected three OS cell lines to detect their mRNA and protein expression levels, and the control group was normal osteoblast hFOB1.19. Compared with normal osteoblast hFOB1.19, the mRNA expression level of DLD and FDX1 were significantly upregulated in 143B cell line, and DLAT was significantly highly expressed in U2OS cell line. In addition, the mRNA expression level of LIPT1 was significantly increased in three OS cell lines HOS, 143B, and U2OS compared to that in hFOB1.19 (**Figure 9A**). The mRNA expression level of AL591767.1 in the 143B cell line was decreased compared with the normal osteoblast hFOB1.19, and AL645608.6 was increased. The mRNA expression level of CARD8-AS1 and AC005041.3 were upregulated in three OS cell lines compared with normal osteoblast hFOB1.19. Besides, compared with normal osteoblast hFOB1.19, the mRNA expression level of AC098487.1 was upregulated in both HOS and U2OS cell lines, and the mRNA expression level of UNC5B-AS1 was upregulated in 143B and U2OS cell lines (**Figure 9B**).

**Figure 10:**
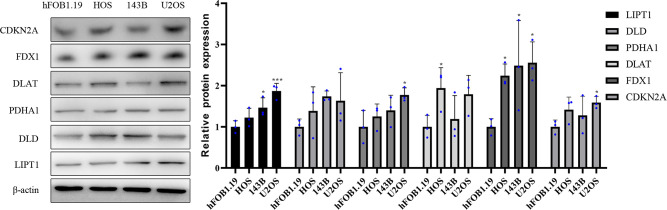
Validation of the protein expression levels of CRGs in OS cell lines. Representative protein grayscale bands. Statistical histogram of grayscale quantification of protein bands. *p < 0.05, ***p < 0.001, each experiment was repeated three times.

Western blotting results showed that compared with normal osteoblast hFOB1.19, the protein expression level of DLAT was significantly highly expressed in HOS cell line, and the protein expression level of PDHA1 and CDKN2A were significantly upregulated in U2OS cell line. in addition, the protein expression level of LIPT1 was significantly elevated in both 143B and U2OS, and the protein expression level of FDX1 was significantly overexpressed in three osteosarcoma cell lines HOS, 143B, and U2OS (**Figure 10**).

**Figure 11:**
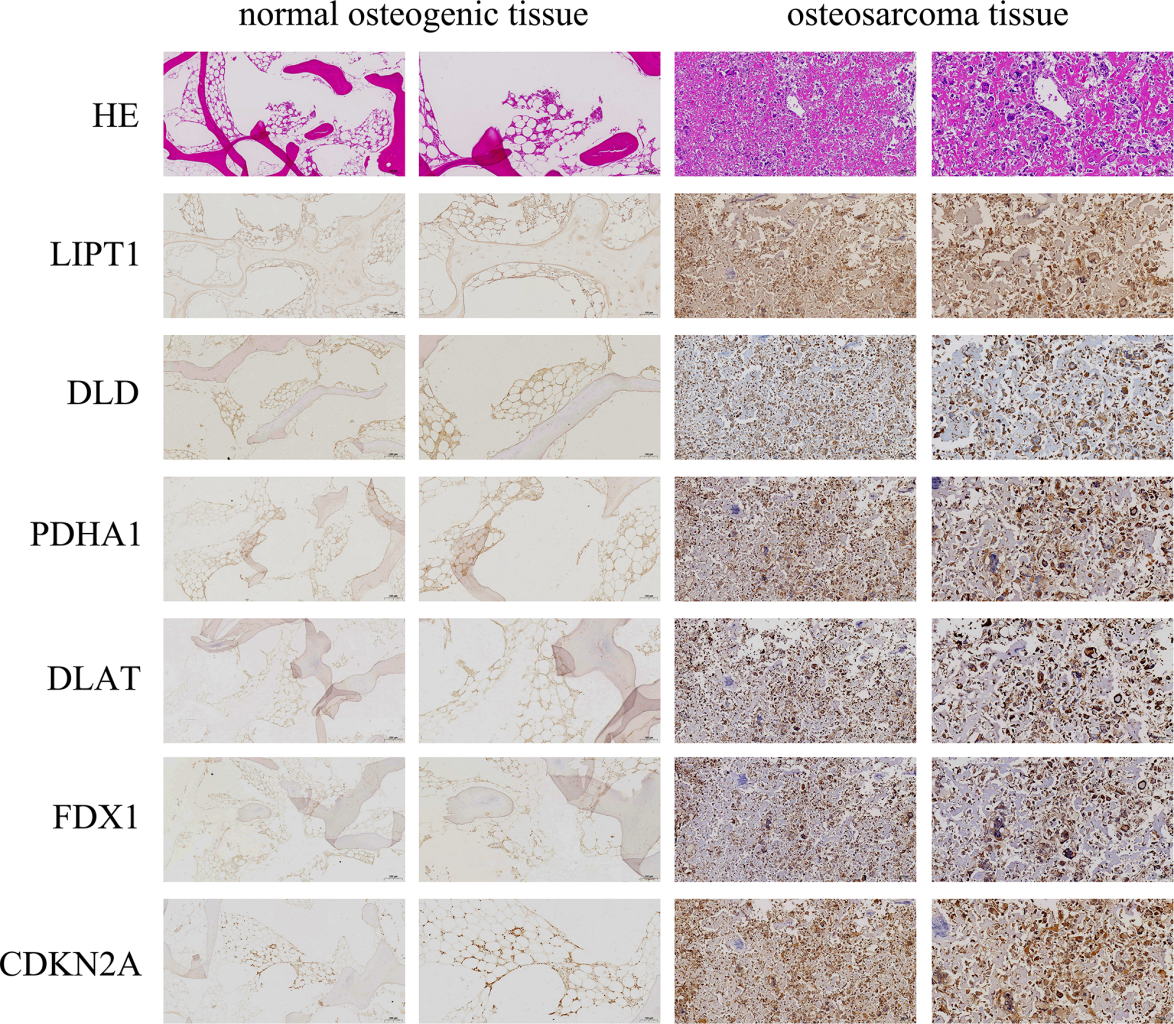
Immunohistochemistry (IHC) staining of OS and normal osteogenic tissue using anti-CRGs antibody and hematoxylin and eosin (HE) staining. Scale bar, 200 μm (left panel) and 100 μm (right panel).

The expression levels of DLAT, DLD, CDKN2A, FDX1, PDHA1, and LIPT1 proteins in OS and normal osteogenic tissues were detected by IHC using the corresponding antibodies and IgG (isotype). The results showed that the expression of DLAT, DLD, CDKN2A, FDX1, PDHA1, and LIPT1 was higher in OS tissues than in normal osteogenic tissues (**Figure 11**).

Overall, it was experimentally verified that the expression of LIPT1, DLAT, and FDX1 at both mRNA and protein levels was significantly elevated in OS cell lines compared with normal osteoblast hFOB1.19. This is consistent with the results of our bioinformatics analysis. LIPT1, DLAT, and FDX1 may be potential targets for the diagnosis and treatment of OS. Besides, the mRNA expression level of AL591767.1 was decreased in OS, and that of AL645608.6, CARD8-AS1, AC005041.3, AC098487.1, and UNC5B-AS1 was upregulated in OS.

## Discussion

Similar to iron, copper is a basic element required for human activities (22). Copper plays an essential role as a cofactor for essential enzymes (22). Copper is a trace element in the human body, and the concentration of intracellular copper ions is maintained at very low levels by active homeostatic mechanisms. Once the threshold is exceeded, copper becomes toxic, leading to cell death (22, 23). Cells dependent on mitochondrial respiration are approximately 1,000 times more sensitive to copper ionophores than glycolytic cells, and mitochondrial antioxidants, fatty acids, and mitochondrial function inhibitors have a significant effect on copper ionophore sensitivity (10). An OS metabolomic study found that the TCA cycle was altered in OS, and both the TCA cycle and glutathione metabolism were downregulated in human OS cancer stem cells (24). When combined with a glutaminase inhibitor (CB-839) and metformin for the treatment of OS, CB-839 limits cell proliferation by forcing dependence on fatty-acid-derived carbons, which reduces aspartate biosynthesis and induces ketosis (25). The combination of CB-839 and metformin not only inhibits OS primary tumor growth but also reduces the risk of OS metastasis (25). In addition, OS cells treated with the combination showed decreased cellular mitochondrial respiration and an overall decrease in glycolysis and TCA cycle function (25). OS is related to the TCA cycle and mitochondrial metabolism. The specific mechanism of cuproptosis is that copper ions induce protein toxic stress responses by binding to fatty acid acylated components in the TCA cycle, inducing cell death (10). Therefore, research on CRLncs that can guide OS prognosis and the immune microenvironment would improve the clinical efficacy of OS.

In this study, a new OS prognosis model was established. Six CRLncs were involved in the construction of the model, including three high-risk CRLncs (AL645608.6, AL591767.1, and UNC5B-AS1) and three low-risk CRLncs (CARD8-AS1, AC098487.1, and AC005041.3). As the expression of high-risk CRLncs in OS increases, the patient risk increases. As the expression of low-risk CRLncs in OS increases, the patient risk decreases. The high- and low-risk groups of the prognostic model differed in their tumor microenvironment. We found that immune cells, such as B cells, macrophages, Th2 cells, and Treg, were significantly downregulated in the high-risk group. Immune functions such as APC co-inhibition, checkpoint, and T-cell co-inhibition were significantly downregulated in the high-risk group. In addition, we identified four drugs with significant sensitivity in the prognostic model (AUY922, bortezomib, lenalidomide, and Z.lle.CHO) that may improve the clinical efficacy of OS. Finally, it was experimentally verified that the expression of LIPT1, DLAT, and FDX1 at both mRNA and protein levels was significantly elevated in OS. Besides, the mRNA expression level of AL591767.1 was decreased in OS, and that of AL645608.6, CARD8-AS1, AC005041.3, AC098487.1, and UNC5B-AS1 was upregulated in OS.

We found that AL645608.6, UNC5B-AS1, and CARD8-AS1 were associated with tumor prognosis, which also reflected to a certain extent the reliability of the results of this study. AL645608.6 is highly correlated with clinical prognosis in patients with acute myeloid leukemia (26). UNC5B-AS1 not only promotes the malignant progression of prostate cancer by competitively binding to caspase-9 (27) but also promotes the proliferation, migration, and invasion of papillary thyroid cancer cell lines (28). CARD8-AS1 is not only a glioma-risk lncRNA (29) but is also significantly associated with overall survival in ovarian cancer (30). The results of studies related to AL591767.1, AC098487.1, and AC005041.3 remain unclear.

The immune environment of OS consists mainly of T lymphocytes and macrophages but also contains other subsets, such as B lymphocytes and mast cells (31). Downregulation of miR-138 expression in OS ameliorates CD4+CXCR5+ follicular helper T cell (Tfh) dysfunction and promotes B-cell differentiation (32). In OS, tumor-associated macrophages not only promote tumor growth and angiogenesis but also inhibit OS metastasis (33). M0 and M2 macrophages derived from the THP-1 human monocyte cell line promoted OS cell migration and invasion more significantly than M1 macrophages did (34). Th2 is also associated with OS metastasis, and the immune-related genes MSR1 and TLR7 associated with macrophages and Th2 can serve as anti-metastatic features of OS (35). Tregs have a potential role in the progression of OS, and the impetus of the antitumor efficacy of anti-PD-1 antibodies in OS mouse models may be decreased numbers of FOXP3+Tregs and increased tumor-infiltrating lymphocytes in the tumor microenvironment (36). A comprehensive analysis of zinc finger protein genes and OS prognosis and the tumor immune microenvironment revealed differences in APC co-inhibition and T-cell-co-inhibition between high- and low-risk groups (37). A study of hypoxic prognostic features associated with OS metastasis and immune infiltration found that immune checkpoints were downregulated in high-risk populations (38).

The combined use of the cyclin-dependent kinase inhibitor SCH727965 (SCH) and heat shock protein 90 inhibitor NVP-AUY922 (AUY922) can induce apoptosis in OS cells (39). SCH and AUY922 may be promising strategies for OS treatment. Bortezomib induces apoptosis and autophagy in OS cells *via* the mitogen-activated protein kinase pathway (40). Bortezomib inhibits OS cell growth and induces apoptosis by inhibiting proteasome (41). The association between lenalidomide and Z.LLNle.CHO and OS remains unclear. The results of this study show that lenalidomide and Z.LLNle.CHO may be potential therapeutic drugs for OS and provide a research direction for improving the clinical efficacy of OS.

However, this study has limitations. First, the sample size of tumors in this study was relatively small. Second, the CRLncs that can guide OS prognosis and the immune microenvironment obtained in this study require further research into the biological function.

## Conclusion

In this study, a new OS prognosis model was established, and six CRLncs were involved in the construction of the model. Through tumor microenvironment and immune-related analyses, we found that these six CRLncs can guide the immune microenvironment of OS. Additionally, we identified four drugs that may have potential efficacy in OS treatment. The results of this study contribute to improving the clinical efficacy and overall survival of patients with OS.

## Data availability statement

The data supporting the results of the study are available from the TCGA database(https://portal.gdc.cancer.gov/) and GEO database (https://www.ncbi.nlm.nih.gov/geo/). The accession number(s) can be found in the article/**Supplementary Material**.

## Ethics statement

This study was reviewed and approved by the Biomedical Research Ethics Committee of HongHui Hospital, Xi’an Jiaotong University (No, 20220600**7**). The patients/participants provided their written informed consent to participate in this study.

## Author contributions

PX and MY designed the study. MY, HZ, and KX analyzed the datasets and interpreted the results. QY downloaded the data. YA and YC provided software support. MY wrote and edited the manuscript. PX provided the foundation and support. All authors contributed to the article and approved the submitted version.

## Funding

This work was financially supported by the National Natural Science Foundation of China (No. 82072432).

## Conflict of interest

The authors declare that the research was conducted in the absence of any commercial or financial relationships that could be construed as a potential conflict of interest.

## Publisher’s note

All claims expressed in this article are solely those of the authors and do not necessarily represent those of their affiliated organizations, or those of the publisher, the editors and the reviewers. Any product that may be evaluated in this article, or claim that may be made by its manufacturer, is not guaranteed or endorsed by the publisher.
